# Central Nervous System-related Conditions and Associated Healthcare Resource Use Among Japanese nmCRPC Patients Based on Retrospective Claims Data

**DOI:** 10.36469/001c.87550

**Published:** 2023-10-31

**Authors:** Dianne A. Ledesma, Jonathan L. Chua, Susan S.H. Tang, Xiu W. Lim

**Affiliations:** 1 Market Access Oncology Bayer Yakuhin, Ltd., Tokyo, Japan; 2 IQVIA Asia Pacific Singapore, Real World Insights, Singapore

**Keywords:** castration-resistant prostate cancer, androgen deprivation therapy, antiandrogen, gonadotropin-releasing hormone analogs, central nervous system-related

## Abstract

**Background:** Japanese patients with prostate cancer are typically treated with primary androgen deprivation therapy (ADT), most commonly administered as a combination of a luteinizing hormone-releasing hormone (LHRH) agonist and an antiandrogen (AA). Since LHRH agonists and AA therapy can be maintained for several years, the long-term effects of these treatments on patients must be carefully considered, including the risk of concomitant central nervous system (CNS) conditions which could affect treatment choices.

**Objective:** To describe CNS-related concomitant conditions during ADT and/or AA treatment and the subsequent healthcare resource utilization in Japanese nonmetastatic castration-resistant prostate cancer (nmCRPC) patients.

**Methods:** Patients diagnosed with nmCRPC and CNS-related conditions while on ADT and/or AA therapy between April 2009 and August 2017 were retrospectively followed up for a maximum of 2 years using a claims database.

**Results:** A total of 455 patients (average age, 78.5 years), were included. The 3 most common concomitant CNS-related conditions were pain (~60% of events), insomnia (~30%), and headache (2%-3%). The frequency of CNS-related conditions in these patients increased approximately threefold after starting AA therapy (before, 969 events; after, 2802). On average, a patient had 10 episodes of concomitant CNS-related conditions in a year. Medical costs did not significantly increase due to CNS-related conditions.

**Discussion:** The most frequently reported CNS-related conditions were pain, insomnia, and headaches. Furthermore, more concomitant CNS-related conditions 1 year after CRPC diagnosis and 1 year after starting AA treatment were recorded.

**Conclusion:** Patients with nmCRPC experience an increase in the frequency of concomitant CNS-related conditions, including pain, insomnia, and headaches, after CRPC diagnosis or starting AA treatment. Future research should explore the causes of this increased frequency.

## INTRODUCTION

Prostate cancer is the second most commonly diagnosed malignancy among males globally and was the most common cancer among males in Japan in 2022.^1^ Japanese patients with prostate cancer are typically treated with primary androgen deprivation therapy (ADT), which can be administered using various methods. The most commonly reported primary ADT treatment for Japanese patients is a combination of luteinizing hormone-releasing hormone (LHRH) agonist and an anti-androgen (AA).^2^ Since LHRH agonists and AA therapy can be maintained for several years, the effects of these treatments on patients in the long term need to be carefully considered. Furthermore, the recent availability of second-generation androgen receptor inhibitors necessitate an examination of the current status of comorbid central nervous system (CNS) conditions that could affect patient care.

CNS-related conditions among prostate cancer patients have gained attention in recent years.^3,4^ A 2020 systematic review by Ryan et al^3^ highlighted the lack of consensus on whether long-term prostate cancer treatment causes an increased risk of CNS comorbid conditions, citing several studies that have found or have not found evidence of increased risk. However, the review cited results from clinical studies showing that the second-generation androgen receptor inhibitors enzalutamide and apalutamide were associated with some CNS-related adverse events, including fatigue (which can interfere with cognitive function), and that abiraterone acetate was associated with a low CNS adverse event profile when compared with enzalutamide, while darolutamide demonstrated a comparable incidence of cognitive disorder in clinical trials compared with ADT alone.^3^

In Japan, a real-world comparison of effects of abiraterone and enzalutamide among men with metastatic castration-resistant prostate cancer (mCRPC) showed differing rates of fatigue among treated patients.^5^ On the other hand, a national database study on prostate cancer patients in Korea concluded that the use of ADT for the treatment of prostate cancer is associated with an increased risk of cognitive dysfunction.^6^ However, other real-world evidence regarding concomitant CNS-related conditions among patients with nonmetastatic CRPC (nmCRPC) have not yet been reported in Japan.

The aim of the study was, therefore, to determine the occurrence of CNS-related conditions among nmCRPC patients in Japan. Second, we aimed to determine the healthcare resource utilization (HRU) and, exploratively, the real-world metastasis-free survival (MFS) of these patients.

## METHODS

### Study Design

This was a retrospective cohort study from a commercially available hospital claims database managed by Medical Data Vision Co., Ltd. (MDV). The database consists of data from April 2008 to August 2018. The full database contains data on about 4.4 million patients in Japan, corresponding to approximately 3% of the total Japanese population. From this database, we selected patients who met the selection criteria described below.

Patients were included into the study if they had been diagnosed with CRPC as defined by Japanese code 8848040 during the index period (April 2009 and August 2017). These patients must also have received at least 1 prescription of medical ADT and AAs within a year prior to diagnosis of CRPC. Medical ADT considered are leuprorelin (L02AE02), goserelin (L02AE03), and degarelix (L02BX02), and AAs included in this study were bicalutamide (L02BB03), flutamide (L02BB01), or enzalutamide (L02BB04). Patients were excluded from the study if they did not have any record of luteinizing hormone-releasing hormone (LHRH)/gonadotropin-releasing hormone agonist (GnRHa) and AA treatment within a year after being diagnosed with CRPC or had no record of any CNS-related conditions within a year after their diagnosis. They were also excluded if they had at least 1 metastatic event corresponding to disease codes C77 (secondary and unspecific malignant neoplasm of lymph node), C79 (secondary malignant neoplasm of other and unspecified sites), and C79.5 (secondary malignant neoplasm of bone and bone marrow) in the year prior to and at CRPC diagnosis, so as to include only nmCRPC patients.

**Figure 1.**
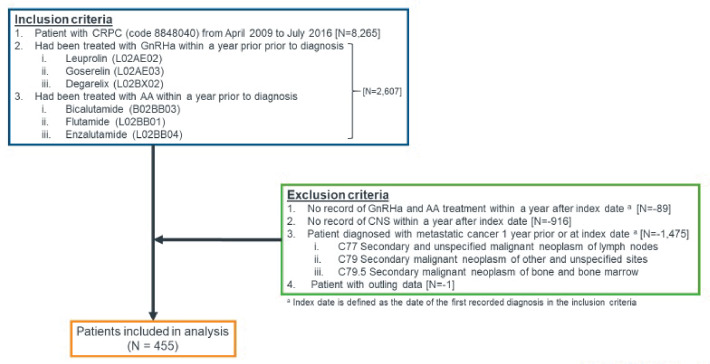
Patient Selection Flow for Cohort Used for All Analyses Except Kaplan-Meier Analysis Abbreviations: CNS, central nervous system; CRPC, castration-resistant prostate cancer.

The cohort of patients who met the above criteria were followed up for 1 year. However, to assess a 2-year MFS, we reduced the cohort size by shortening the index period to between April 2009 and August 2016. A 2-year MFS follow-up was chosen based on literature-based evidence reporting that, of those patients with no metastases present at diagnosis of CRPC, 33% could expect to develop them within 2 years.^7^ The patient selection flow is illustrated in **Figure 1**: 455 patients were included in the analysis, of which 313 patients in the reduced cohort size were included for the MFS analysis.

**Figure 2.**
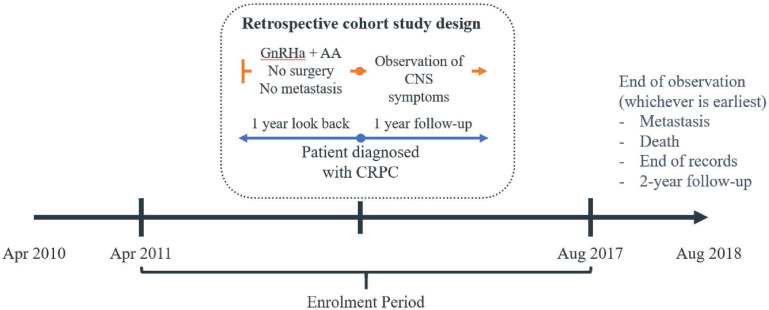
Study Observation Period of Patients 1 Year Pre-Post CRPC Therapy Abbreviations: AA, anti-androgen; CNS, central nervous system; CRPC, castration-resistant prostate cancer; GnRHa, gonadotropin-releasing hormone agonist.

To identify nmCRPC patients with concomitant CNS-related conditions and the HRU associated with concomitant CNS-related conditions against GnRHa and AA therapy, the study observation period considered was set to 1 year pre-post CRPC therapy (**Figure 2**).

To compare CNS-related conditions pre-post AA therapy, we observed the occurrence of diseases 1 year pre-post first AA therapy (figure not shown).

### Study Outcome Measures

Three outcome measures were included: the occurrence of concomitant CNS conditions in nmCRPC and their associated treatment history, HRU, and, exploratively, the length of MFS among these patients.

CNS-related conditions were extracted using the *International Classification of Diseases, Tenth Revision* (ICD-10) as reference for conditions listed under “CNS-related conditions,” as previously cited.^4^ CNS conditions included any claim relating to amnesia or memory impairment, anxiety, ataxia, cognitive disorders, confusion, convulsions, disturbance in attention, dizziness, falls, fatigue, hallucinations, headaches, insomnia, pain, paresthesia, seizures, weakness, narcolepsy and cataplexy, and other CNS disorders. The **Appendix** lists the CNS conditions considered in this study and their associated ICD-10 codes.

For the second outcome measure, only the 3 metastatic ICD-10 codes used in the patient selection process were considered as metastatic outcomes for the MFS analysis (C77, C79, and C79.5).

Lastly, healthcare costs were calculated in Japanese yen (JPY) and were presented for total direct medical costs, medication costs with CRPC treatment as a subset, diagnostic costs, hospitalization including intensive care unit (ICU) admission costs, and outpatient costs. These were assessed during the 1-year follow-up period. For CNS treatment–related costs, therapies that were included in the cost calculations were those listed as prescribed treatment for those indications in the MDV database. Although there were many possible therapeutic options for each CNS condition, only treatments that appeared in the database factored in the cost calculations. The costs in the database already reflected the Healthcare Fee System and NHI Drug Price List for the applicable year in Japan.

### Statistical Analysis

We performed descriptive analyses and stratified the results by treatments taken, concomitant CNS-related condition, and whether the condition occurred before and after the first diagnosis of CRPC or the first prescription of AA treatment.

To protect the anonymity of patients, the dates in the MDV dataset were partially masked. Only the month and year of each entry was available. Hence, we used a 12-month addition to calculate a 1-year follow-up period. All costs were calculated in JPY. These analyses were carried out using Python (Python Software Foundation. Python Language Reference, version 3.7.3. Available at http://www.python.org).

A logistic regression analysis was used to study the risk factors for the 3 most common concomitant CNS-related conditions. Age and comorbidities were included in the model as confounding variables. Additionally, the number of CNS symptom events and treatments taken were used as explanatory variables. The most parsimonious model was selected. To study the variables associated with total direct medical costs, a generalized linear model with gamma distribution and log link was used. This was because the outcome of interest, healthcare cost, had a long right-tail distribution. Most statisticians and health economists recommend this model instead of log transformation of the data as, in most cases, it does not normalize the variable. All variables were kept in this model to facilitate comparison between the 3 CNS-related conditions. For Kaplan-Meier analysis, a different cohort was used, utilizing patients who had at least 2 years of post-CRPC diagnosis data. The event of interest was metastatic events based on ICD-10 codes C770, C790, and C795. The date of death or the end of the 2-year follow-up period or the date of the last data point available, whichever came earliest, was used to censor patients. All patients were analyzed in the same group. For these analyses, R version 3.6.1 (R Core Team [2019]. *R: A Language and Environment for Statistical Computing.* R Foundation for Statistical Computing, Vienna, Austria. https://www.R-project.org/) was used.

### IRB Approval and Informed Consent Statement

The conduct of this study was approved by a Central IRB (Health Outcomes Research Institute; Approval No. 2019-3) in Tokyo, Japan. This study used only anonymized retrospective data from a commercially available secondary database source (MDV) using only existing materials.

## RESULTS

### Descriptive Characteristics of the Study Population

**Table 1** shows the demographic characteristics of the cohort on the index date and at the end of 1 and 2 years of follow-up. The average age of patients with nmCRPC diagnosis on the index date was 78.5 years (range, 54-98 years). The mean Charlson Comorbidity Index (CCI) score was 1.8 on the index date and increased by 0.1 with each year of follow-up.^8^ The most common comorbidity at CRPC diagnosis was mild liver disease (29.8%), while the most common comorbidity that developed during the follow-up period was metastatic solid tumor (from 12.5% to 37.7% at 1-year follow-up, probably pertaining to the development of metastatic CRPC). Prostate-specific antigen (PSA) levels increased over time, but because only 15% of patients had PSA test results, this might not be an accurate summary of the cohort. Lastly, over 20% of patients progressed to the metastatic stage, and about 15% died within the first year of CRPC diagnosis.

**Table 1.** Demographic Characteristics of nmCRPC Patients With Concomitant CNS Disease

**Variable**	**At CRPC Diagnosis**	**At 1-⁠Year Endpoint**	**At 2-⁠Year Endpoint**
	**(N = 455)**	**(N = 455)**	**(N = 313)**
Year of CRPC diagnosis			
No. of patients with data	455	–	313
Mean (SD)	–	–	–
Median (IQR)	2016 (2015, 2016)	–	2015 (2015, 2016)
Range	2014, 2017	–	2014 , 2016
Age (y)			
No. of patients with data	455	455	313
Mean (SD)	78.5 (7.6)	79.3 (7.6)	79.5 (7.6)
Median (IQR)	79 (73-84)	80 (74-85)	80 (75-85)
Range	54-98	55-99	55-98
Charlson Comorbidity Index			
Mean (SD)	1.8 (1.8)	1.9 (1.9)	2 (2)
Median (IQR)	1 (0 , 3)	2 (1 , 4)	2 (1 , 4)
Range	0-9	0-9	0-9
Charlson comorbidities			
Myocardial infarction	22 (4.8%)	23 (5.0%)	13 (4.2%)
Congestive heart failure	65 (14.3%)	87 (19.1%)	65 (20.8%)
Peripheral vascular disease	37 (8.1%)	41 (9.0%)	29 (9.3%)
Cerebrovascular disease	75 (16.4%)	95 (20.8%)	63 (20.1%)
Dementia	9 (2.0%)	16 (3.5%)	15 (4.8%)
Chronic pulmonary disease	74 (16.2%)	92 (20.2%)	64 (20.4%)
Rheumatoid disease	13 (2.9%)	16 (3.5%)	14 (4.5%)
Peptic ulcer disease	114 (25.0%)	163 (35.7%)	112 (35.8%)
Mild liver disease	136 (29.8%)	152 (33.3%)	115 (36.7%)
Diabetes without end-organ damage	57 (12.5%)	76 (16.7%)	60 (19.2%)
Diabetes with end-organ damage	25 (5.5%)	31 (6.8%)	22 (7.0%)
Hemiplegia	3 (0.7%)	6 (1.3%)	3 (1.0%)
Moderate or severe renal disease	31 (6.8%)	37 (8.1%)	28 (8.9%)
Any malignancy except basal cell cancer of skin	99 (21.7%)	110 (24.1%)	75 (24.0%)
Moderate liver disease	4 (0.9%)	6 (1.3%)	4 (1.3%)
Metastatic solid tumor	57 (12.5%)	172 (37.7%)	149 (47.6%)
HIV/AIDS	0 (0.0%)	0 (0.0%)	0 (0.0%)
PSA level			
No. of patients with data	65	67	43
Mean (SD)	14.6 (24.3)	228.7 (1310.3)	172 (362.8)
Median (IQR)	6.4 (3.64-⁠11.18)	4.4 (0.97-⁠40.06)	20.4 (2.027-⁠206.19)
Range	0-124.4	0-10715.4	0-2115.1
Treatment used prior to CRPC diagnosis^a^			
Leuprorelin	261 (57.2)	–	–
Goserelin	185 (40.6)	–	–
Degarelix	81 (17.8)	–	–
Bicalutamide	320 (70.2)	–	–
Flutamide	250 (54.8)	–	–
MFS	455 (100%)	349 (76.5%)	211 (67.4%)
Death	0 (0%)	65 (14.3%)	81 (25.9%)

^a^Only treatments that were indicated for prostate cancer prior to CRPC are included.

Abbreviations: CNS, central nervous system; CRPC, castration-resistant prostate cancer; IQR, interquartile range; MFS, metastasis-free survival; nmCRPC, nonmetastatic castration-resistant prostate cancer; PSA, prostate-specific antigen.

### Proportion of Patients with Concomitant CNS-related Conditions Before and After CRPC Diagnosis and AA Treatment

There were 4822 concomitant CNS diseases recorded. The most common concomitant CNS diseases were pain, insomnia, and headaches, affecting 76.7%, 34.5%, and 7.0% of patients, respectively. These 3 CNS diseases accounted for over 90% of concomitant CNS disease events, with the bulk being pain (65.7%). On average, each patient experienced about 10 CNS symptoms during the 1-year follow-up period. Seizures were also observed (**Table 2**). Pain conditions retrieved included low back pain, lumbar region; chest pain, unspecified; other chronic pain (including “continuous pain,” “cancer pain,” “breakthrough cancer pain”); and unspecified pain (classified in the claims database only as “pain, unspecified”).

**Table 2.** One-Year Period Prevalence of CNS Conditions After CRPC Diagnosis and After First AA Treatment

**Variables**	**1 Year After CRPC**		**1 Year After AA Treatment**	
	**No. (%) of Patients**	**No. (%) of Events**	**No. (%) of Patients**	**No. (%) of Events**
Total	455 (100)	4822 (100)	297 (100)	2802 (100)
Amnesia or memory impairment	1 (0.2)	7 (0.1)	2 (0.7)	11 (0.4)
Anxiety	12 (2.6)	90 (1.9)	6 (2.0)	41 (1.5)
Ataxia	1 (0.2)	2 (0.0)	0 (0.0)	0 (0.0)
Cognitive disorders	14 (3.1)	32 (0.7)	5 (1.7)	20 (0.7)
Confusion	0 (0.0)	0 (0.0)	0 (0.0)	0 (0.0)
Convulsions	0 (0.0)	0 (0.0)	0 (0.0)	0 (0.0)
Disturbance in attention	0 (0.0)	0 (0.0)	0 (0.0)	0 (0.0)
Dizziness	17 (3.7)	83 (1.7)	11 (3.7)	57 (2.0)
Falls	0 (0.0)	0 (0.0)	0 (0.0)	0 (0.0)
Fatigue/asthenia	6 (1.3)	36 (0.7)	5 (1.7)	27 (1.0)
Hallucinations	0 (0.0)	0 (0.0)	0 (0.0)	0 (0.0)
Headaches	32 (7.0)	137 (2.8)	13 (4.4)	55 (2.0)
Insomnia	157 (34.5)	1166 (24.2)	111 (37.4)	787 (28.1)
Pain	349 (76.7)	3167 (65.7)	223 (75.1)	1749 (62.4)
Paraesthesia	5 (1.1)	8 (0.2)	3 (1.0)	4 (0.1)
Seizures	18 (4.0)	89 (1.8)	10 (3.4)	50 (1.8)
Weakness	3 (0.7)	5 (0.1)	1 (0.3)	1 (0.0)

Pain reported in the database were low back pain, lumbar region; chest pain, unspecified; other chronic pain (including “continuous pain,” “cancer pain,” “breakthrough cancer pain”); and unspecified pain (classified in the claims database only as “pain, unspecified”).

Abbreviations: CNS, central nervous system; CRPC, castration-resistant prostate cancer.

When we compared the number of patients with concomitant CNS-related conditions before and after CRPC diagnosis and AA treatment, we observed that rarely do concomitant CNS-related conditions stop once they begin for patients in the cohort. Among patients without previous CNS-related conditions, 157 (34.5%) developed said conditions after CRPC diagnosis and 162 (35.6%) after the start of AA treatment; 298 (65.5%) experienced CNS-related conditions prior to CRPC diagnosis or AA treatment, and 138 (30.3%) continued to experience these post-CRPC and post-AA treatment (**Supplementary Figure S1, Supplementary Tables S1 and S2**). The median time to the first CNS-related condition from the time of CRPC diagnosis was about 6 months. This was noted to be the same median time regardless of the nature of the CNS symptom (table not shown).

### Period Prevalence of Concomitant CNS-related Conditions Before and After AA Treatment

There was a rise in the number of patients, partly due to selection criteria of the cohort. Nevertheless, the number of CNS-related conditions rose threefold after AA treatment. This was most visible in the 3 most commonly recorded CNS-related conditions: pain (before, 543 events [56%]; after, 1749 [62.4%]), insomnia (before, 312 events [32.2%]; after, 787 [28.1%]), and headaches (before, 31 events [3.2%]; after, 55 [2%]). Combined, these 3 CNS-related conditions accounted for about 90% of the total number of events. Our results also show that most patients will report almost 10 concomitant CNS-related conditions in the 1 year after AA treatment begins.

### Occurrence of Seizures

Seizures were diagnosed in 10 patients, with 4 patients having previously experienced seizure events prior to the start of AA therapy and continuing to experience seizures after AA therapy was started, while 6 patients were newly diagnosed with seizure events within the year after AA therapy was started. Due to the very small sample size of patients with seizure events, our analyses are exploratory at best. However, patients diagnosed with seizure tended to be older (median age, 82.5 years; interquartile range [IQR], 79.75-83.75) compared with the overall population in this study (median age, 79 years; IQR, 73-84) (**Supplementary Table S3**). Patients also had no recorded diagnosis of CNS metastatic disease (ICD-10 G96.9), although they tended to have other CNS-related conditions such as pain and insomnia, at around the same time as the seizures (median 5 months, IQR 2-9).

### Risk Factors for Concomitant CNS-related Conditions

We then investigated the possible risk factors for concomitant CNS-related conditions: pain, insomnia, and headaches. These included age, comorbid conditions, treatment types, and prior CNS-related conditions. Results showed that prior pain incidence increased a patient’s odds of pain recurrence significantly (odds ratio [OR] = 72.2; 95% confidence interval [CI] = 27.1-232, *p* < .0001) while prior incidence of insomnia (OR = 0.043, 95% CI = 0.043-0.2, *p* < .0001) and headaches (OR = 0.174, 95% CI = 0.037-0.794, *p* < .0239) were protective for pain incidence during follow-up. A similar trend was also observed for insomnia, where prior incidence of insomnia had an extremely large OR (OR = 1470, 95% CI = 281-14 816, *p* < .0001) while prior pain diseases were protective (OR = 0.208, 95% CI = 0.092-0.434, *p* < .0001) and decreased the odds of future concomitant insomnia events substantially. Prior headaches were also very good predictors of headache recurrence (OR = 914, 95% CI = 100-28 857, *p* < .0001), but no other concomitant CNS-related condition showed the protective effect (**Supplementary Table S4**).

### Healthcare Resource Utilization of the Study Population

**Table 3** shows the cost associated to HRU. The mean total direct medical cost was about 4 million JPY (US $28 000) but could reach in excess of 56 million JPY (US $390 000) in a year. Most of the cost was attributable to medication. Onaverage, medicines cost approximately 3 million JPY (US $21 000) over a 1-year period, of which about 2 million JPY was for CRPC medication. Consequently, other healthcare resources accounted for a smaller proportion of the total direct medical costs. Claims for laboratory tests and diagnostic imaging were 0.14 and 0.1 million JPY (US $900), respectively. Hospitalization costs were higher at approximately 1.6 million JPY per patient (US $11 000). Patients were on average hospitalized twice during the follow-up year. ICU events were rare: only 6 patients were admitted to the ICU, and their mean cost was about 0.6 million JPY (US $4000). Patients had regular outpatient clinic visits, averaging 20 outpatient clinic visits each during the follow-up period.

**Table 3.** Treatment Cost (in Million JPY) for HRU During 1-Year Follow-up

**Variable**	**n**	**Total (N = 455)**
Age (y), mean (SD)	455	79.3 (7.6)
Total direct medical costs (million JPY)	455	
Mean (SD)		3.99 (3.21)
Median (IQR)		3.83 (2.35-5.11)
Range		0.26-56.36
Medication costs (million JPY)	455	
No. of patients		455
No. of events		93622
Total costs (million JPY)		
Mean (SD)		2.99 (3.01)
Median (IQR)		2.77 (1.34-4.21)
Range		0.19-56.17
CRPC costs (million JPY)	455	
Mean (SD)		2.02 (1.47)
Median (IQR)		1.65 (0.75-3.02)
Range		0.07-6.38
CNS costs (million JPY)	329	
Mean (SD)		0.024 (0.05)
Median (IQR)		0.01 (0.00-0.02)
Range		0.0-0.48
Diagnostic costs (million JPY)		
Laboratory costs	454	
Mean (SD)		0.13 (0.10)
Median (IQR)		0.11 (0.08-0.16)
Range		0.01-1.06
Imaging costs	404	

Abbreviations: HRU, health resource utilization; ICU, intensive care unit; IQR, interquartile range; JPY, Japanese yen. The total cost for CNS-related conditions was not noted to significantly increase overall costs of CRPC treatment.

However, Japan has universal health insurance coverage, as well as a high cost-benefit medical insurance system that can offset patients’ direct out-of-pocket costs; hence, the costs here do not directly reflect how much a patient actually pays for treatment.

### Predictors of Higher Medical Costs

Next, we quantified the effect of concomitant CNS-related conditions on medical costs to patients. The final model consisted of 2 of the most commonly seen concomitant CNS-related conditions in this data set (pain and insomnia) and CRPC treatments. Neither the number of concomitant CNS-related conditions nor CRPC treatment duration led to noticeable differences in the total direct medical cost.

### Survival Curves of nmCRPC Patients

Our exploratory analysis using Kaplan-Meier curves showed that 102 of 313 patients had a metastatic event in the 2-year follow-up (~30%), of which about 80% of metastatic events occurred within the 12 months after the index date; only 19 additional patients progressed to metastasis in the second year (**Supplementary Figure S2**). The average time to metastatic event in the 2-year follow-up was 8.3 months, and 6.2 months for events that occurred in the first 12 months. It is worthwhile to note that this estimate is only for nmCRPC patients with CNS-related conditions, which may differ from nmCRPC patients without these conditions (beyond the scope of this study). We also did not qualify whether patients were considered high- or low-risk nmCRPC patients, as only limited PSA data were available (and no imaging results available) in the database.

## DISCUSSION

We described the prevalence of concomitant CNS-related conditions, HRU, and time to metastatic event in nmCRPC patients to provide the Japanese context of the ongoing discussion in the literature of CNS-related conditions in prostate cancer patients.

We found that the most often reported CNS-related conditions were pain, insomnia, and headaches, in that order. Furthermore, this group of patients experienced more concomitant CNS-related conditions 1 year after CRPC diagnosis and 1 year after starting AA treatment. The analysis suggests that recurrence of concomitant CNS-related conditions was commonplace, which is consistent with current literature regarding the higher incidence or risk of fatigue, headaches, and insomnia with AA therapy.^3,9^ However, concomitant CNS-related conditions contributed only a small percentage of the direct medical costs to patients, whereas CRPC treatments accounted for the majority. As we used a medical claims database as the source for this study, this may imply underreporting of CNS disease or underprescribing for diagnosed concomitant CNS-related conditions. For example, Onishi et al^10^ showed that the rate of prescription of opioids for both acute and chronic pain in Japan is very low compared with the United States. It is also notable that the most common CNS-related conditions in these patients (pain, headaches) can be treated by generic drugs and/or over-the-counter medications.

The pain conditions retrieved in our study included low back pain, lumbar region; chest pain, unspecified; other chronic pain (including “continuous pain,” “cancer pain,” “breakthrough cancer pain”); and unspecified pain (classified in the claims database only as “pain, unspecified”). Interestingly, this result is highlighted by qualitative literature on nmCRPC patients’ most commonly reported symptoms being “pain” as well.^11^ The sources of spinal pain in prostate cancer are thought to be varied (eg, activity- or movement-related, multifactorial, malignant), and our findings also reveal such varied types of pain.^12^ Furthermore, pain is more commonly associated with metastatic prostate cancers due to metastasis to the bone, and, more importantly, is reported to be predictive of overall survival in prostate cancer, so the presence of pain in what we define as patients with nmCRPC in this study may point to the need for closer monitoring (such as timely diagnostic imaging) of patients with nmCRPC to ensure that possible metastasis is found as early as possible and patients are given the most appropriate treatment.^13^

A literature review by Ryan et al^3^ found that fatigue was the common CNS-related condition reported for ADTs. Pilon et al^4^ also reported fatigue as the most common CNS event, with pain the next most common, but our study showed a higher proportion of patients with recorded pain events and fewer recorded fatigue/asthenia events across all kinds of treatments (LHRH or AAs). As our data come from a claims database, this may imply underreporting of fatigue by physicians in Japan, as evidenced by Ooki et al,^5^ whose study showed that Japanese physicians underreported fatigue by 56.7% compared with what cancer patients had reported.

The incidence of seizures in patients on higher doses of some AAs has led to suggestions that they can cross the blood-brain barrier, and by extension other treatments may also have the same potential, although their ability to penetrate may vary significantly.^3,6^ Our study showed an increase in the occurrence of seizures either 1 year post-CRPC and/or 1 year post-AA treatment. Specifically, seizures were noted in 10 patients, but the results are inconclusive due to the very small sample. However, older age and the presence of other CNS-related conditions such as pain, insomnia, and headache as a possible constellation of symptoms that occur among these patients may need further exploration. It has been previously stipulated that a previous history of seizures increases the risk of further seizure events in patients taking AAs^7^; however, our study also shows a number of newly diagnosed seizure patients after starting AA treatment. Future studies could explore the possible risks for seizure occurrence among patients with CRPC.

In contrast, several studies have also shown that androgens were protective against neurodegeneration.^8,9^ Low androgen levels were associated with loss of synapse in the hippocampus, increases in amyloid depositions, and changes to the neurotransmission in the prefrontal cortex.^9^ Tan et al^8^ found that andropause led to memory problems but concluded that the effect was subtle and potentially unmeasurable.

Despite a plausible biological explanation for a causal relationship between ADT and CNS conditions among prostate cancer patients, it is still unclear if there is a causal relationship.^3^ Several studies have shown clear associations between ADT and CNS conditions,^10,11^ but many others did not find any associations.^12–14^ Even though our descriptive analysis was suggestive of an association, we did not find an association in the regression models. There are 2 possible explanations for this: first, many confounders were not accounted for and, second, individual variation and susceptibility may play a greater role. This study was not able to account for several potential variables, including genetic variation, societal factors, and physical activity, which could be confounders, given that it is a retrospective and medical records–based study.^3^ In particular, aging-associated hormonal changes have been postulated to confound the results,^3^ but in our study, age was neither significant nor had large effect sizes for all models. Hence, further research is needed to clarify the role of ADTs in the development and frequency of concomitant CNS-related conditions over prolonged exposure.

The model findings also showed that prior concomitant CNS-related conditions were risk factors for future occurrence. This was not unexpected, as a similar pattern is seen for many other diseases, including heart diseases like stroke and heart attacks. It merely indicates that the causal pathway of these concomitant CNS-related conditions remained active and therefore may have led to recurrence.

We showed that the average annual costs of treatment were substantial, in the range of 3 million to 5 million JPY (US $20 000-$34 000), which is almost the same range as the reported annual income of Japanese CRPC patients.^11^ Mahlich et al calculated comparable costs when looking at cost after chemotherapy started in CRPC patients, while a model by Kunisawa et al showed that hospitalization accounted for higher costs and that treatment costs were much lower.^15,16^ A previous study found that for all CRPC patients, the mean annual total direct medical costs were much lower than those in this study, and medication accounted for most of the costs. A possible cause of the discrepancy is the source of the data.^17^ The Japanese healthcare system does grant a copayment rate that benefits elderly individuals, such as those in this study. For example, patients over 70 years old will shoulder 10% to 30% of the total medical costs depending on their income. Furthermore, they could also utilize the High-Cost Medical Care Benefit system to cover costs exceeding certain thresholds. Our data showed, on average, an additional cost of 20 000 JPY (US $194) for the treatment of CNS-related conditions. This could be due to patients not being prescribed medications for the most common diseases reported (headaches, insomnia, pain), or patients obtaining medications as readily available OTC drugs, hence not reflected in our data source.

Lastly, the number of patients who progressed to metastasis was sizeable, and the time to metastatic event was short—about 8 months on average. The median MFS rates reported by Hussain et al,^18^ using an international cohort, was shown to be just over 3 years. Although our study had a follow-up period of 2 years, our results were comparable, where approximately 30% of patients had a metastatic event within 24 months.

The study has several limitations. First, as a medical claims database, the population within this database may not be representative of the general Japanese population, nor would it be similar to clinical trial populations of androgen receptor inhibitors, where CNS-related adverse events were closely monitored. Second, diagnosis of CRPC and CNS-related conditions is dependent on clinicians accurately and timely reporting, and in some cases, patients declaring their symptoms. Hence, the patient numbers could be underestimated. Third, our sample size is relatively small, and only patients with concomitant CNS-related conditions were selected. Fourth, the follow-up period was short, and patients frequently changed treatments during the follow-up period, thereby complicating the relationship between treatment and CNS disease. Finally, patients who changed hospitals and for whom data were lost in the 1-year follow-up period would also have been omitted, resulting in possible bias in the results.

## CONCLUSIONS

Patients with nmCRPC with concomitant CNS-related conditions have greater morbidity 1 year after CRPC diagnosis and 1 year after starting AA treatment, with increased frequency of concomitant CNS-related conditions, although we observed that they had similar MFS as nmCRPC patients in other studies. The presence of pain and the short time to metastatic event within our observation period highlights the need for diagnostic imaging to be conducted at the proper time to be able to potentially improve patients’ prognoses. The presence of concomitant CNS-related conditions should be carefully considered in treatment decision-making for nmCRPC patients, as nmCRPC treatment duration is relatively long, and these occurrences will affect patients’ quality of life.

### Author Contributions

D.L. conceived of the study and its design, led the coordination of the study, and helped draft the manuscript. J.C., X.W.L., and S.T. participated in the development of the study design, carried out the statistical analysis, and helped draft the manuscript.

### Disclosures

J.C., X.W.L., and S.T. were employees of IQVIA, Real World Insights at the time of conduct of this study, which was commissioned by Bayer Yakuhin, Ltd. D.L. is a full-time employee of Bayer Yakuhin, Ltd.

## Supplementary Material

Supplementary Online Material
