# Supplementary material for: Central Nervous System-related Conditions and Associated Healthcare Resource Use Among Japanese nmCRPC Patients Based on Retrospective Claims Data
Source: J Health Econ Outcomes Res. 2023 Oct 31;10(2):91–9. doi: 10.36469/001c.87550 (PMC10621534; doi:10.36469/001c.87550)
Supplement: Supplementary Online Material [file jheor_2023_10_2_87550_185642.pdf]

JHEOR

Journal of Health Economics and Outcomes Research

## Online Supplementary Material

Central Nervous System–related Conditions and Associated Healthcare Resource Use Among Japanese nmCRPC Patients Based on Retrospective Claims Data. *JHEOR*. 2023;10(2):91-99. doi:10.36469/jheor.2023.87550

**Figure S1: Number of Patients with CNS-related Conditions (a) 1 Year Before and After CRPC Diagnosis, and (b) 1 Year Before and After First Anti-androgen Treatment**

**Figure S2: Kaplan-Meier Curve of Metastatic Events for All Patients**

**Table S1: Demographic Characteristics of nmCRPC Patients With Seizure (n = 10)**

**Table S2: Period Prevalence of Concomitant CNS-related Conditions 1 Year Before and After First AA Treatment**

**Table S3: Demographic Characteristics of nmCRPC Patients With Seizure (n = 10)**

**Table S4: Risk Factors for Concomitant CNS Disease Occurrence During CRPC Treatment in the Cohort**

This supplementary material has been provided by the authors to give readers additional information about their work.

This is an open-access article distributed under the terms of the Creative Commons Attribution 4.0 International License (CCBY-4.0). View this license's legal deed at http://creativecommons.org/licenses/by/4.0 and legal code at http://creativecommons.org/licenses/by/4.0/legalcode for more information.

**Figure S1.** Patients with CNS-related Conditions (**a**) 1 Year Before and After CRPC Diagnosis, and (**b**) 1 Year Before and After First AA Treatment

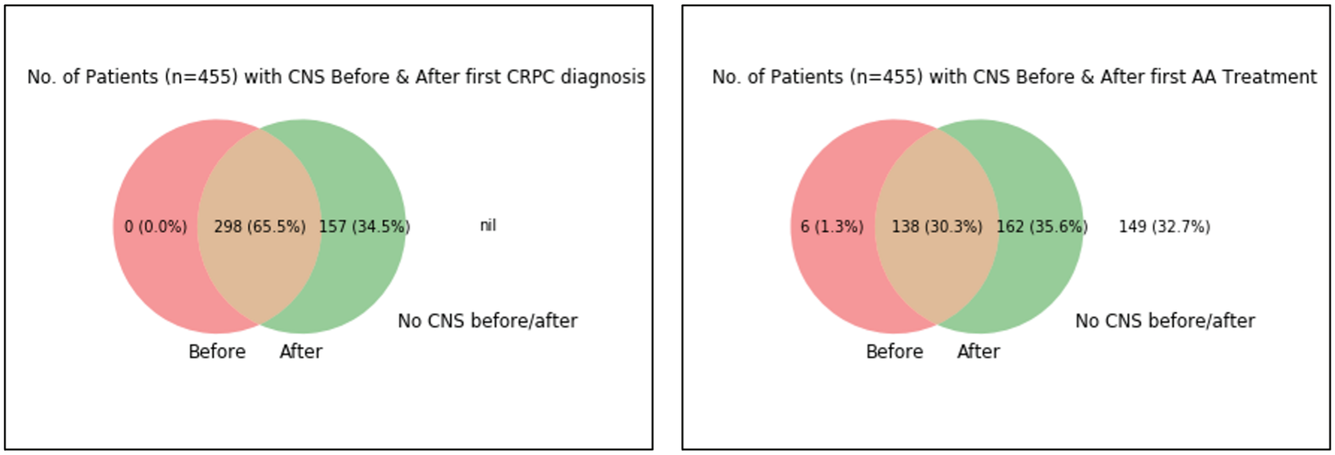


a

b

Abbreviations: AA, anti-androgen; CNS, central nervous system; CRPC, castration-resistant prostate cancer.

**Figure S2.** Kaplan-Meier Curve of Metastatic Events for All Patients

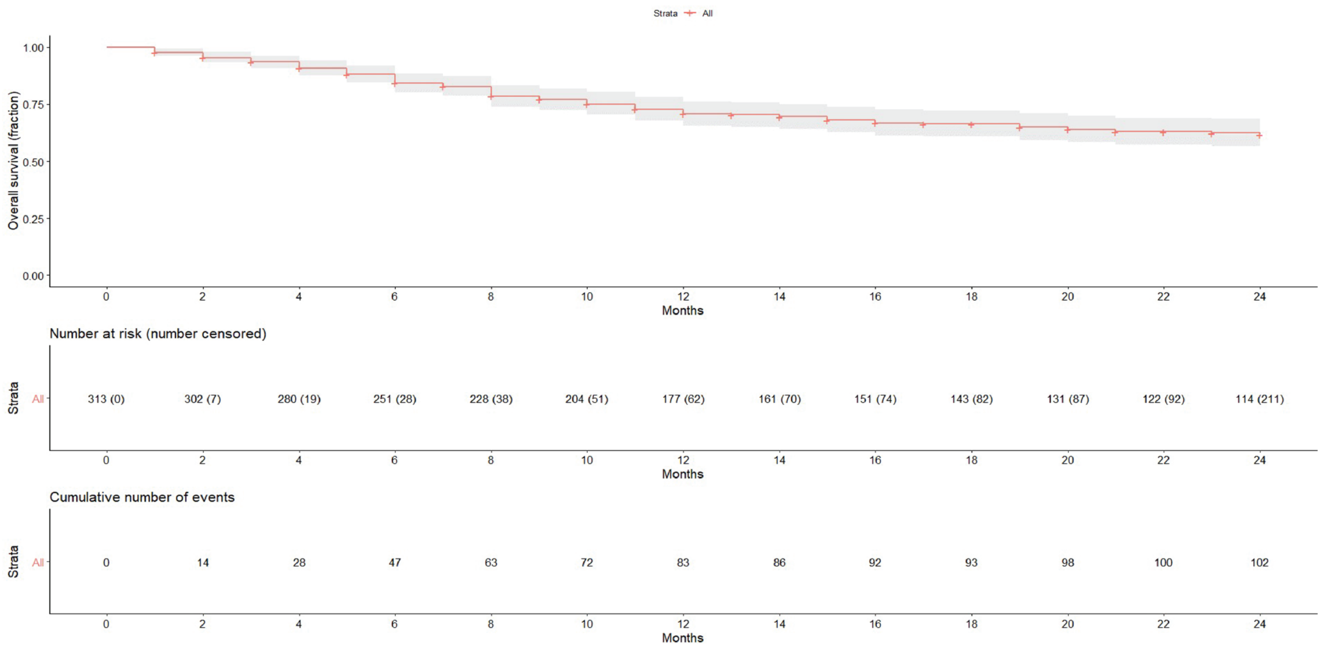

**Table S1.** Period Prevalence of Concomitant CNS-related Conditions 1 Year Before and After CRPC Diagnosis, and 1 Year Before and After First AA Treatment in the Cohort

| **Variable** | **1 Year Before CRPC Diagnosis** | | **1 Year After CRPC Diagnosis** | | **1 Year Before AA Treatment** | | **1 Year After AA Treatment** | |
|---|---|---|---|---|---|---|---|---|
| | **No. of Events** | **%** | **No. of Events** | **%** | **No. of Events** | **%** | **No. of Events** | **%** |
| Total | 2792 | 100 | 4822 | 100 | 969 | 100 | 2802 | 100 |
| Amnesia or memory impairment | 11 | 0.40 | 7 | 0.10 | 11 | 1.10 | 11 | 0.40 |
| Anxiety | 62 | 2.20 | 90 | 1.90 | 27 | 2.80 | 41 | 1.50 |
| Ataxia | 0 | 0.00 | 2 | 0.00 | 2 | 0.20 | 0 | 0.00 |
| Cognitive disorders | 16 | 0.60 | 32 | 0.70 | 4 | 0.40 | 20 | 0.70 |
| Confusion | 0 | 0.00 | 0 | 0.00 | 0 | 0.00 | 0 | 0.00 |
| Convulsions | 0 | 0.00 | 0 | 0.00 | 0 | 0.00 | 0 | 0.00 |
| Disturbance in attention | 0 | 0.00 | 0 | 0.00 | 0 | 0.00 | 0 | 0.00 |
| Dizziness | 67 | 2.40 | 83 | 1.70 | 13 | 1.30 | 57 | 2.00 |
| Falls | 0 | 0.00 | 0 | 0.00 | 0 | 0.00 | 0 | 0.00 |
| Fatigue asthenia | 17 | 0.60 | 36 | 0.70 | 9 | 0.90 | 27 | 1.00 |
| Hallucinations | 0 | 0.00 | 0 | 0.00 | 0 | 0.00 | 0 | 0.00 |
| **Headaches** | **87** | **3.10** | **137** | **2.80** | **31** | **3.20** | **55** | **2.00** |
| **Insomnia** | **839** | **30.10** | **1166** | **24.20** | **312** | **32.20** | **787** | **28.10** |
| **Pain** | **1619** | **58.00** | **3167** | **65.70** | **543** | **56.00** | **1749** | **62.40** |
| Paresthesia | 2 | 0.10 | 8 | -0.20 | 3 | 0.30 | 4 | 0.10 |
| Seizures | 69 | 2.50 | 89 | 1.80 | 14 | 1.40 | 50 | 1.80 |
| Weakness | 3 | 0.10 | 5 | -0.10 | 0 | 0.00 | 1 | 0.00 |

Abbreviations: AA, anti-androgen; CRPC, castration-resistant prostate cancer.

**Table S2.** Period Prevalence of Concomitant CNS-related Conditions 1 Year Before and 1 Year After First AA Treatment

| **Variable** | **1 Year Before AA Treatment** | | **1 Year After AA Treatment** | |
|---|---|---|---|---|
| | **No. (%) of Patients** | **No. (%) of Events** | **No. (%) of Patients** | **No. (%) of Events** |
| Total | 144 (100) | 969 (100) | 297 (100) | 2802 (100) |
| Amnesia or memory impairment | 2 (1.4) | 11 (1.1) | 2 (0.7) | 11 (0.4) |
| Anxiety | 4 (2.8) | 27 (2.8) | 6 (2.0) | 41 (1.5) |
| Ataxia | 1 (0.7) | 2 (0.2) | 0 (0.0) | 0 (0.0) |
| Cognitive disorders | 1 (0.7) | 4 (0.4) | 5 (1.7) | 20 (0.7) |
| Confusion | 0 (0.0) | 0 (0.0) | 0 (0.0) | 0 (0.0) |
| Convulsions | 0 (0.0) | 0 (0.0) | 0 (0.0) | 0 (0.0) |
| Disturbance in attention | 0 (0.0) | 0 (0.0) | 0 (0.0) | 0 (0.0) |
| Dizziness | 3 (2.1) | 13 (1.3) | 11 (3.7) | 57 (2.0) |
| Falls | 0 (0.0) | 0 (0.0) | 0 (0.0) | 0 (0.0) |
| Fatigue/asthenia | 1 (0.7) | 9 (0.9) | 5 (1.7) | 27 (1.0) |
| Hallucinations | 0 (0.0) | 0 (0.0) | 0 (0.0) | 0 (0.0) |
| Headaches | 7 (4.9) | 31 (3.2) | 13 (4.4) | 55 (2.0) |
| Insomnia | 52 (36.1) | 312 (32.2) | 111 (37.4) | 787 (28.1) |
| Pain | 102 (70.8) | 543 (56.0) | 223 (75.1) | 1749 (62.4) |
| Paraesthesia | 2 (1.4) | 3 (0.3) | 3 (1.0) | 4 (0.1) |
| Seizures | 4 (2.8) | 14 (1.4) | 10 (3.4) | 50 (1.8) |
| Weakness | 0 (0.0) | 0 (0.0) | 1 (0.3) | 1 (0.0) |

Abbreviation: AA, anti-androgen; CNS, central nervous system.

**Table S3.** Demographic Characteristics of nmCRPC Patients With Seizure (n = 10)

| | **Seizure-Diagnosed Patients: Both Periods** | | | **Newly Diagnosed Seizure Patients After First AA Treatment** | | | **Seizure-Diagnosed Patients (All)** | | | **Overall Population** | | |
|---|---|---|---|---|---|---|---|---|---|---|---|---|
| **Variable** | **At CRPC Diagnosis (n = 4)** | **At 1-Year Endpoint (n = 4)** | **At 2-Year Endpoint (n = 1)** | **At CRPC Diagnosis (n = 6)** | **At 1-Year Endpoint (n = 6)** | **At 2-Year Endpoint (n = 3)** | **At CRPC Diagnosis (n = 10)** | **At 1-Year Endpoint (n = 10)** | **At 2-Year Endpoint (n = 4)** | **At CRPC Diagnosis (n = 455)** | **At 1-Year Endpoint (n = 455)** | **At 2-Year Endpoint (n = 313)** |
| Age (y) | | | | | | | | | | | | |
| No. of patients with data | 4 | 4 | 1 | 6 | 6 | 3 | 10 | 10 | 4 | 455 | 455 | 313 |
| Mean (SD) | 79.5 (7) | 80.5 (7) | 84 (NA) | 82 (5.1) | 82.5 (5) | 80.3 (5.1) | 81 (5.7) | 81.7 (5.6) | 81.2 (4.6) | 78.4 (7.6) | 79.3 (7.6) | 79.5 (7.6) |
| Median (IQR) | 82.5 (78.75-83.25) | 83.5 (79.75-84.25) | 84 (84-84) | 82.5 (79.75-84.5) | 83 (79.75-85.5) | 79 (77.5-82.5) | 82.5 (79.75-83.75) | 83.5 (79.75-84.75) | 81.5 (78.25-84.5) | 79 (73-84) | 80 (74-85) | 80 (75-85) |
| Range | 69-84 | 70-85 | 84-84 | 74-89 | 75-89 | 76-86 | 69-89 | 70-89 | 76-86 | 54-98 | 55-99 | 55-98 |
| Charlson Comorbidity Index score | | | | | | | | | | | | |
| Mean (SD) | 2.6 (2.6) | 2.8 (2.8) | 6 (NA) | 1.5 (1.5) | 1.9 (1.9) | 2 (2) | 1.9 (1.9) | 2.1 (2.1) | 2.2 (2.2) | 1.8 (1.8) | 1.9 (1.9) | 2 (2) |
| Median (IQR) | 3 (1.5-4.5) | 3.5 (1.5-5.25) | 6 (6-6) | 2 (2-3.5) | 3.5 (2.25-4.75) | 3 (2-4) | 2 (2-4) | 3.5 (2-5) | 4 (2.5-5.25) | 1 (0-3) | 2 (1-4) | 2 (1-4) |
| Range | 0-6 | 0-6 | 6-6 | 1-5 | 1-6 | 1-5 | 0-6 | 0-6 | 1-6 | 0-9 | 0-9 | 0-9 |

Abbreviations: CRPC, castration-resistant prostate cancer; IQR, interquartile range; nmCRPC, nonmetastatic castration-resistant prostate cancer.

**Table S4.** Risk Factors for Concomitant CNS Disease Occurrence During CRPC Treatment in the Cohort

| Variable | Pain: Pseudo $R^2 = 0.554$ | | | Insomnia: Pseudo $R^2 = 0.715$ | | | Headache: Pseudo $R^2 = 0.204$ | | |
|---|---|---|---|---|---|---|---|---|---|
| | OR | 95% CI | *p* Value | OR | 95% CI | *p* Value | OR | 95% CI | *p* Value |
| Comorbidity | | | | | | | | | |
| Myocardial infarction | 4.32 | 0.883, 23.23 | .077 | 1.42 | 0.211, 7.916 | .7016 | 0.11 | 0, 6.387 | .4814 |
| Congestive heart failure | 0.374 | 0.126, 1.084 | .0708 | 1.1 | 0.318, 3.407 | .8753 | 0.727 | 0.077, 4.124 | .7459 |
| Peripheral vascular disease | 1.62 | 0.442, 6.273 | .4721 | 0.179 | 0.023, 1.094 | .0806 | 2.68 | 0.294, 18.25 | .3363 |
| Cerebrovascular disease | 1.24 | 0.504, 3.207 | .642 | 0.359 | 0.084, 1.231 | .1285 | 1.04 | 0.188, 4.205 | .9636 |
| Dementia | 217 | 2.834, 336725 | .0957 | 34.8 | 3.127, 621.563 | .0061 | 0.133 | 0, 5.575 | .4042 |
| Chronic pulmonary disease | 0.678 | 0.284, 1.646 | .3827 | 1.38 | 0.504, 3.584 | .5174 | 0.744 | 0.126, 3.154 | .712 |
| Connective tissue disease | 0.231 | 0.037, 1.424 | .1048 | 1.25 | 0.107, 10.369 | .8462 | 4.95 | 0.237, 42.439 | .1951 |
| Peptic ulcer disease | 0.601 | 0.283, 1.275 | .1816 | 1.72 | 0.765, 3.769 | .1782 | 2.72 | 0.816, 8.583 | .0905 |
| Mild liver disease | 1.49 | 0.729, 3.151 | .2838 | 0.772 | 0.332, 1.687 | .5297 | 1.54 | 0.47, 4.565 | .4517 |
| Diabetes without end-organ damage | 0.452 | 0.169, 1.227 | .1142 | 0.659 | 0.183, 2.03 | .4926 | 1.46 | 0.284, 5.947 | .618 |
| Diabetes with end-organ damage | 1.11 | 0.207, 6.271 | .9004 | 5.14 | 0.78, 28.058 | .0676 | 0.311 | 0.005, 5.028 | .4951 |
| Hemiplegia | 161000 | 0, NA | .9928 | 2.59E-06 | NA, 1.469e+69 | .9924 | 5.61E-07 | NA, 1.050e+94 | .9969 |
| Moderate or severe renal disease | 1.4 | 0.423, 4.819 | .5862 | 3.74 | 0.953, 14.025 | .0515 | 2.17 | 0.266, 11.669 | .4083 |
| Any malignancy except basal cell cancer of skin | 0.888 | 0.416, 1.932 | .7613 | 1.41 | 0.587, 3.254 | .425 | 1.17 | 0.31, 3.817 | .8006 |
| Moderate liver disease | 0.377 | 0.014, 18.328 | .5806 | 0.889 | 0.004, 62.948 | .9694 | 2.96E-07 | NA, 2.968e+75 | .996 |
| Metastatic solid tumor | 0.465 | 0.176, 1.232 | .1208 | 1.47 | 0.518, 3.846 | .4431 | 0.501 | 0.041, 2.739 | .4972 |
| Age (y) | 0.967 | 0.926, 1.009 | .1268 | 1.02 | 0.977, 1.073 | .3266 | 1 | 0.935, 1.077 | .9446 |
| Prior CNS events (Y/N) | | | | | | | | | |
| Anemia or memory impairment | 4E-09 | NA, 1.285e+69 | .9902 | 0.00555 | 0, 22.407 | .6301 | 65.8 | 0.499, 53675.426 | .1238 |
| Anxiety | 0.231 | 0.026, 1.915 | .1669 | 3.42 | 0.15, 42.454 | .3524 | 8.97E-08 | NA, 8.765e+36 | .993 |
| Cognitive disorders | 0.0341 | 0, 9.569 | .2754 | 0.0384 | 0.001, 1.892 | .1101 | 14.1 | 0.056, 812.041 | .2372 |
| Dizziness | 0.519 | 0.09, 3.314 | .4692 | 0.509 | 0.013, 6.973 | .682 | 0.453 | 0.009, 6.898 | .6293 |
| Fatigue/esthenia | 2.08 | 0.066, 95.6 | .6905 | 97900000 | 0, NA | .9838 | 5.08E-09 | NA, 4.088e+153 | .9947 |
| Headache | 0.174 | 0.037, 0.794 | .0239 | 0.49 | 0.039, 3.408 | .5271 | 914 | 100.138, 38857 | <.0001 |
| Insomnia | 0.0958 | 0.043, 0.2 | <.0001 | 1470 | 281.625, 14816 | <.0001 | 0.216 | 0.024, 1.039 | .0966 |
| Pain | 72.2 | 27.123, 232.762 | <.0001 | 0.208 | 0.092, 0.434 | .0001 | 0.369 | 0.106, 1.091 | .0865 |
| Paresthesia | 4.14E-09 | NA, 5.695e+202 | .9936 | 7.26E-07 | NA, 1.106e+204 | .9953 | 6.79E-08 | NA, Inf | .998 |
| Seizures | 0.407 | 0.063, 3.038 | .3533 | 0.136 | 0.004, 2.323 | .2315 | 0.498 | 0.003, 9.95 | .722 |
| Weakness | 161000000 | 0, NA | .9876 | 0.0873 | 0, 729.206 | .8413 | 1.13E-06 | NA, 1.562e+111 | .9976 |

Abbreviations: CI, confidence interval; CNS, central nervous system; CRPC, castration-resistant prostate cancer; NA, not applicable; OR, odds ratio.
